# Gemcitabine Combination Nano Therapies for Pancreatic Cancer

**DOI:** 10.3390/pharmaceutics11110574

**Published:** 2019-11-04

**Authors:** Kamalika Samanta, Saini Setua, Sonam Kumari, Meena Jaggi, Murali M. Yallapu, Subhash C. Chauhan

**Affiliations:** 1 Department of Pharmaceutical Sciences and Center for Cancer Research, University of Tennessee Health Science Center, Memphis, TN 38163, USA; ksamanta@uthsc.edu (K.S.); ssetua@uthsc.edu (S.S.); skumari@uthsc.edu (S.K.); 2 Department of Immunology and Microbiology, Institute for Cancer Immunotherapy, School of Medicine, University of Texas Rio Grande Valley, McAllen, TX 78503, USA; meena.jaggi@utrgv.edu

**Keywords:** Gemcitabine, chemotherapy, nanoparticles, drug resistance, combination therapy

## Abstract

Pancreatic cancer is one of the deadliest causes of cancer-related death in the United States, with a 5-year overall survival rate of 6 to 8%. These statistics suggest that immediate medical attention is needed. Gemcitabine (GEM) is the gold standard first-line single chemotherapy agent for pancreatic cancer but, after a few months, cells develop chemoresistance. Multiple clinical and experimental investigations have demonstrated that a combination or co-administration of other drugs as chemotherapies with GEM lead to superior therapeutic benefits. However, such combination therapies often induce severe systemic toxicities. Thus, developing strategies to deliver a combination of chemotherapeutic agents more securely to patients is needed. Nanoparticle-mediated delivery can offer to load a cocktail of drugs, increase stability and availability, on-demand and tumor-specific delivery while minimizing chemotherapy-associated adverse effects. This review discusses the available drugs being co-administered with GEM and the limitations associated during the process of co-administration. This review also helps in providing knowledge of the significant number of delivery platforms being used to overcome problems related to gemcitabine-based co-delivery of other chemotherapeutic drugs, thereby focusing on how nanocarriers have been fabricated, considering the modes of action, targeting receptors, pharmacology of chemo drugs incorporated with GEM, and the differences in the physiological environment where the targeting is to be done. This review also documents the focus on novel mucin-targeted nanotechnology which is under development for pancreatic cancer therapy.

## 1. Introduction

Pancreatic cancer (PanCa) is the fourth highest cause among cancer-related deaths (56,770 people, 29,940 men, and 26,830 women) in 2018 in the United States. It was also estimated that about 23,800 men and 21,950 women were diagnosed with this disease. PanCa accounts for only ~3% of all cancers but it contributes ~7% of cancer deaths. PanCa accounts for very poor overall median survival, i.e., 5 to 6 months. The 5-year survival rate for patients with pancreatic cancer is only 6 to 8% [1]. This type of malignant neoplasm occurs from the mucosa of the pancreas ductal epithelium which could be due to pancreatic intraepithelial neoplasia (PanIN), mucinous cystic neoplasm (MCN), intraductal papillary mucinous neoplasm (IPMN), and invasive ductal adenocarcinoma. Pancreatic ductal adenocarcinoma (PDAC) is considered as the most common pathological type of pancreatic cancer and contributes to 94% of pancreatic cancers [2]. The literature has revealed that patients with pancreatic cancer were often observed with 90% KRAS mutations, 50 to 80% inactivating mutations in CDKN2A, SMAD4, and TP53 [3]. Diagnosis of pancreatic cancer at an early stage is highly challenging due to the lack of medically known urgent symptoms. Surgical resection of tumors is only possible in the case of localized tumors in the pancreas [3]. Unfortunately, by the time of diagnosis, many patients experience either a locally advanced stage or distant metastasis, and then become ineligible candidates for surgical operative procedures. This happens in a majority of patients, thus there is a high priority to manage disease progression while promoting quality of lifespan. In such a scenario, chemotherapy along with surgery is a potentially useful and highly preferred curative approach. Patients with advanced pancreatic cancer refractory status to first-line therapy have a dismal prognosis and limited therapeutic options, with only one option pertaining to nanoliposome irinotecan in combination with fluorouracil and folic acid which was approved by FDA based upon results of a phase III NAPOLI-1 study [4]. Clinical activity of Gemcitabine has been progressively seen in non-small cell lung cancer, head and neck cancer, colon cancer, breast cancer, and pancreatic cancer. According to the report generated by the NCI (National Cancer Institute) studies are still ongoing in monitoring Gemcitabine as a potential inhibitor of cancer. In 833 locations, GEM is given in combination with Pemetrexed and it is believed that the addition of ramucirumab and pembrolizumab will enhance patient survival by inhibiting tumor growth [5]. Another ongoing study on PANOVA-3 is being carried out on 12 locations; PANOVA-3 is in a phase III clinical trial which altogether consists of tumor treating fields (TTFs) which involves use of specific frequencies of the electric field which cause disruption of cell division. In this trial, TTFs are used in combination with GEM and nab-PTX for treating PanCa [6]. Therefore, in this review, we present an in-depth analysis of the various combinational studies of Gemcitabine and evaluate their superior roles with respect to treatment options. This review also provides possible advanced nanotechnology combination options for future investigations.

## 2. Gemcitabine—A Gold Standard Chemotherapeutic Agent for Pancreatic Cancer

Chemotherapy is expected to provide substantial local control and prolong survival. However, there is no efficient and standard treatment for advanced and metastatic pancreatic cancer. Gemcitabine, a deoxycytidine nucleoside analog (2′-deoxy-2′,2′-difluorocytidine; dFdC), has shown a broad spectrum of anticancer activity against various tumors, including pancreatic, lung, and breast cancers. GEM activity depends on its entry into cells and immediate phosphorylation by deoxycytidine kinase (DCK) takes place which results in monophosphate and diphosphate (dFdCDP) [7,8]. The anticancer activity results from diphosphate due to the inhibition of ribonucleotide reductase. Triphosphate metabolite (dFdCTP) is another active metabolite of GEM which can get incorporated into DNA. It locks DNA polymerase and causes DNA chain termination which is required for DNA synthesis [9].

Gemcitabine as a chemotherapeutic agent is not instantaneous due to severe systemic side effects. For example, a phase I trial with a maximum dose of 9 mg/m^2^ five times daily led to fever and fatal hypotension while a phase II trial (150 mg/m^2^ twice weekly) showed flu, fever, rigors, and malaise as its side effects which led towards the withdrawal of GEM [10]. Dissatisfactory results obtained from the failed trials led towards another set of phase I trials with once a week or once every two weeks drug infusions (790 mg/m^2^/week) which resulted in positive activity with minor side effects [11]. Subsequent positive outcomes of clinical trials of GEM led to the approval of this molecule as a potential anticancer agent by the FDA (08/04/2011).

GEM is considered a gold-standard treatment for pancreatic cancer. It has also exhibited a broad spectrum of anticancer activity against various cancers. However, chemoresistance is one of the leading problems associated with this drug. To overcome the side effects caused by GEM, it has been formulated in other forms for effective administration and therapeutic outcome. Problems associated with GEM therapy also opened platforms to administer drugs such as OXA, CIS, and other chemotherapeutic drugs to be administered with GEM, which are being successfully used at the clinical level, as described later in the article.

### 2.1. Gemzar

Gemzar^®^ is a hydrochloride salt of GEM being produced by Eli Lilly which is extensively used against pancreatic cancer both as a single therapeutic agent and in combination with many other anticancer agents. Although it is not immediately life-threatening but Gemzar induces numerous side effects such as pale skin, easy bruising or bleeding, numbness, tingly feeling, weakness, nausea, vomiting, stomach upset, diarrhea, constipation, headache, swelling in hands/ankles/feet, skin rash, drowsiness, and hair loss [12].

The major drawback associated with the delivery of Gemzar is the formation of inactive metabolite difluorodeoxyuridine in the presence of enzyme deoxycytidine deaminase which exists abundantly in the liver and blood. Deoxycytidine monophosphatase causes deamination and conversion of Gemzar to its diphosphate and triphosphate forms. This conversion causes rapid clearance and a reduced half-life of the drug up to 15 min [13]. 

The efficacy of Gemzar as a first-line treatment for pancreatic cancer was done by conducting clinical studies and comparing Gemzar with 5-Fluoro-Uracil. During the experiment, clinical data were obtained from 126 patients suffering from pancreatic cancer with significant symptoms. The drug dose given was either GEM 1000 mg/m^2^ weekly × 7 followed by 1 week of rest, then weekly × 3 every 4 weeks thereafter (for 63 patients), or 5-FU 600 mg/m^2^ once weekly (for 63 patients). The expected result was to alleviate at least one parameter associated with symptoms which could help in increasing patient survival rates without causing additional side effects. A significant change in survival rate was observed in 23.8% of patients treated with GEM compared with just 4.8% clinical benefits in patients under the influence of 5-FU, which concluded the comparatively high therapeutic activity of GEM [14].

### 2.2. HPMA Copolymer-Based Gemcitabine Formulation

Poly(*N*-(2-hydroxypropyl)methacrylamide) (PHPMA) is a copolymer which is widely used to formulate various anticancer drugs. HPMA copolymer formulation provides prolonged GEM by utilizing enhanced permeability and retention effects which localizes the drug at the tumor site [15]. The PHPMA-GEM formulation was made in two forms, A-Gemcitabine (A-GEM) and B-Gemcitabine (B-GEM). A-GEM drug-polymer conjugate was made using uncleavable amino hexanoic acid spacers whereas B-GEM was formulated using glycyl-phenyl-alanyl-leucyl-glycine (GFLG) spacers with an ability to get cleaved by lysosomal cysteine protease cathepsin. HPMA formulations were proposed to be used in conjunction with radiation therapy. Particularly, B-GEM formulation demonstrated a 100% drug release within 6 h in the presence of radiotherapy and showed much more efficacy than GEM alone [16].

### 2.3. Gemlip

GEM suffers from enzymatic inactivation and gets converted to its primary metabolite 2′,2′-difluoro-deoxyuridine (dFdU) in presence of enzyme cytidine deaminase being present abundantly in the liver and plasma [17]. Gemlip is a liposome-based GEM formulation (hydrogenated egg phosphatidylcholine/cholesterol) with an equal amount of drug inside and outside the liposomal shell. Such a composition allows GEM to be present in a constant portion between the vesicle cores and the aqueous space. This formulation enhanced the half-life of the drug up to 13 h, protecting it from deamination, increased therapeutic efficacy up to 35-fold more than GEM alone, and decreased the maximal tolerable dose from 360 to 6–9 mg/kg [18].

### 2.4. Co-Delivery of Gemcitabine

The use of single chemotherapeutic agents has shown limitations caused by their poor stability and bioavailability, drug-resistance, and high systemic toxicity. Co-delivery of dual or multiple/cocktail drugs have come up with synergistic therapeutic action and minimized side effects [19]. Though GEM is preferred as a first-line treatment for pancreatic cancer, its enzymatic plasma deaminase decreases its half-life to 8 to 17 min [20]; that demands an increase in drug-dose which would lead towards probable side effects. Common side effects associated with GEM include: black/tarry stools or blood in urine/stools, bleeding gums, swelling of the face and other parts of body, vision issues, chest pain, cough, diarrhea, dizziness, fever, headache, burning, crawling, itching, numbness, difficulty in swallowing, joint pain, pale skin, paralysis, ulcers, sore throat, trouble sleeping, tiredness/weakness, and weight loss [21].

**Figure 1:**
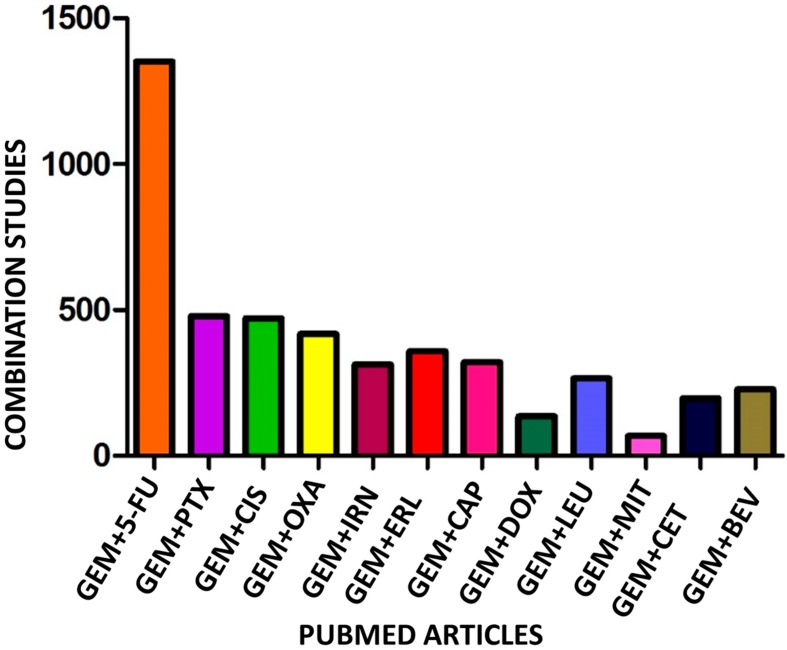
Peer-reviewed publications, book chapters, comments, and reviews, related to Gemcitabine co-administration with various other drugs to treat pancreatic cancer. The number of publications was obtained from archived data in PubMed (up to December 2018).

A significant number of literatures is available in PubMed suggesting that co-administration of GEM with other drugs has vast clinical significance in treating pancreatic cancer (Figure 1).

**Figure 2:**
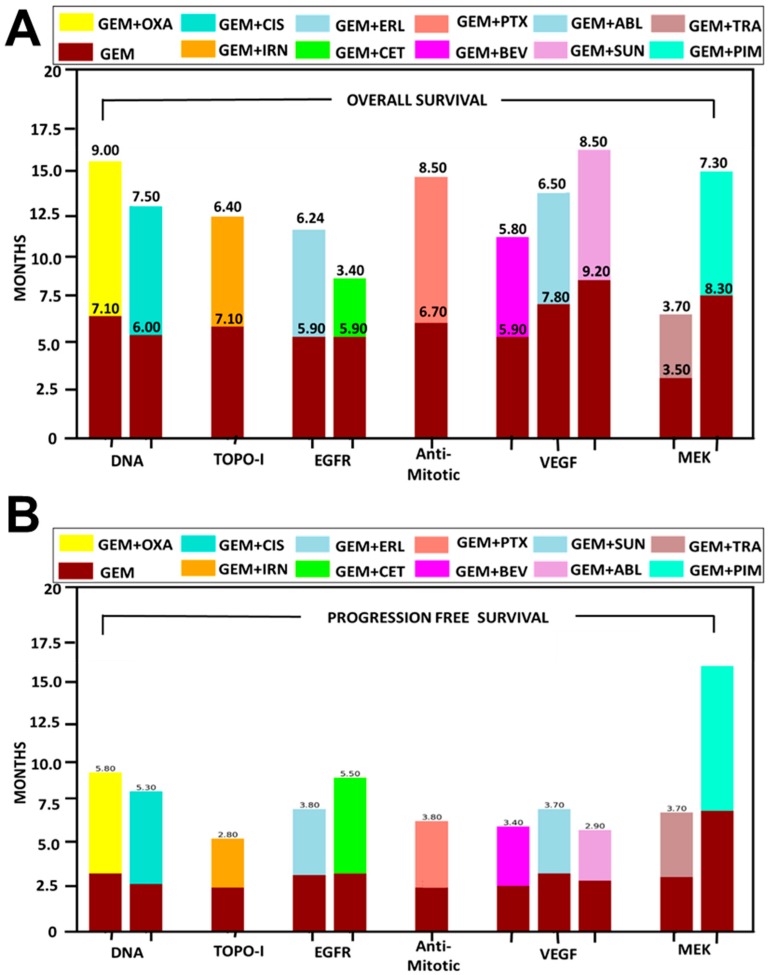
A detailed analysis of GEM co-administration outcomes with other drug candidate in terms of (**A**) overall survival and (**B**) progression-free survival of patients through various clinical trials.

Detailed review reports discussing the superiority and complexity of regiments to increase the overall survival of pancreatic patients are shown in Figure 2A. In addition, Figure 2B suggests a number of clinical studies supporting combinatorial therapeutic interventions of GEM with other drugs are promising and can be utilized as newer therapeutic regimens for progression-free survival of PanCa.

Altogether, co-administration of GEM with other drugs (Figure 2) depict several marginal survival benefits while it may further introduce complexity of toxicity and procedures. Therefore, an additional and safe way to deliver these GEM drug combinations using nanoparticle-mediated delivery may serve as a new tool to improve clinical benefits of combination therapy. The nanomedicine approach enables tumor-specific delivery of the payload while offering minimized systemic or off-target effects. Therefore, our later sections are aimed at presenting and discussing recent advances in delivering nanoparticle-mediated multiple drugs to pancreatic tumors in order to achieve superior therapeutic benefits.

## 3. Nano Formulations Involved in Gemcitabine Co-Administration and Co-Delivery

**Figure 3:**
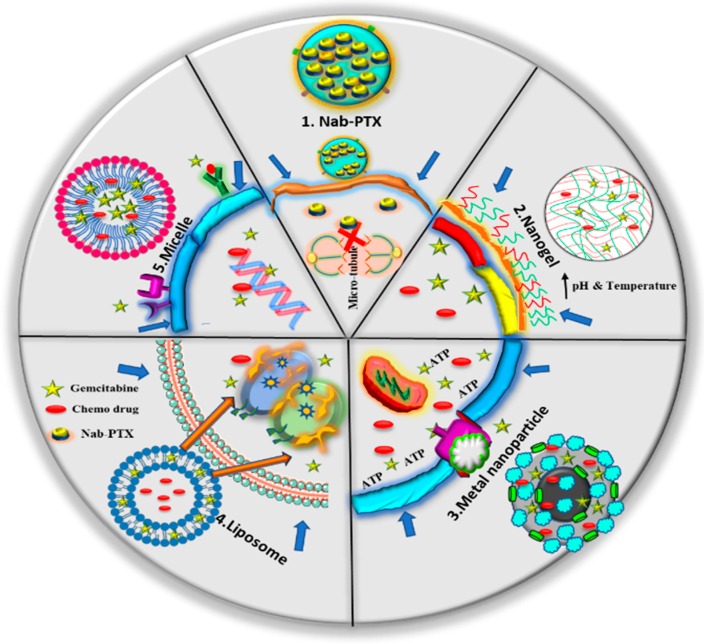
Different nanoparticle mechanisms of delivering therapeutic agents: (1) nab-PTX causes cell cycle arrest by inhibiting polymerization of microtubules during mitosis within recipient cells [34], (2) nanogel releases the drug within the cell in stimulation to suitable pH and temperature as the pH and temperature of cancer cells are higher than normal cells [35], (3) metal-based magnetic nanoparticle checks drug resistance by inhibiting and blocking the P-gp drug efflux mechanism when they are taken up by cancer cells via micropinocytosis, which further obstructs P-gp because of the large size of the nanoparticles [36], (4) liposomes cross the lipid bilayer to target the cancer cells directed by biomarkers aberrantly expressed in PanCa [37], and (5) a micelle encapsulated drug acts as a ligand to target receptors which are specific to GEM-based co-therapy [38].

Gemcitabine co-administration with various chemotherapeutic drugs have shown significant effects and positive outcome at clinical level (Figure 2) and has also been published extensively [22,23,24]. However, a common problem was observed due to the difference in their profiles such as hydrophilicity-to-hydrophobicity, pharmacokinetics, toxicity, and mode of actions when administered together with other drugs. The distance of the target site often leads towards significant drug loss and problems related to drug release in a time-controlled manner. These factors cause a change in drug rate kinetics which does not provide the expected drug effect. Therefore, it is important to design drug delivery models which preserve the integrity of the drug and its release at the targeted site of action. Thus, successful delivery of dual/multi drugs to pancreatic tumors is an encouraging path for developing effective therapeutic regimens. Identifying an effective delivery carrier that does not introduce systemic toxicity is highly warranted. At this point, nanoparticles come into existence as carriers for drug molecules [25]. In general, depending on their use, nanoparticles are considered to be 1–200 nm in dimension. In the medicinal field, nanoparticles are often designed in a way that drug/bio-molecules can be incorporated within and do not get degraded at undesired sites, which can help the drug to reach the targeted site of action more efficiently. Often, nanoparticles can improve the circulation time of the loaded therapeutic molecules and improve its residence at the tumor site through leaky vasculature by enhancing permeation and retention (EPR) effects [26,27,28,29]. Many approved (paclitaxel albumin-bound nanoformulation (Abraxane^®^, Celgene) doxorubicin liposomal formulation (Doxil, currently with Caelyx), micellar formulation of PTX (Genexol-PM, Samyang Biopharm)) and clinically evaluated nanoformulations obey EPR delivery characteristics [30]. Upon reaching the targeted site, nanoparticles internalize (the process of endocytosis, phagocytosis, and pinocytosis) and communicate with cancerous cells [31]. This characteristic avails the drug at a tumor specific site which avoids the leakage of the drug into normal tissue and decreases the incidence of adverse side effects [32]. Cellular internalization attained by nanoparticles is generally ligand-mediated, i.e, expression of certain proteins or receptor are aberrant in cancer cells which are utilized during the designing of nanoparticles to concentrate the drug at the targeted site, avoiding off-target effects. Certain receptors which are overexpressed in cancer cells, such as EGFR, GPCR, FR, TFR, etc. [31], can be used to target more precisely. Thus this section aims to delineate various options of developing therapeutic nanoformulations for dual/multiple therapeutic molecule delivery to PanCa (Figure 3). This can be achieved by various means, including but not limited to, altering the chemical composition, design/sequence, and decoration with targeted motifs and so on [33]. 

Various synthetic approaches, both topdown and bottom-up, of nanocarrier development via nanoprecipitation, double emulsion, solvent evaporation, self-assembly, layer-by-layer approaches, polymer micellization, and gelation are suitable with the appropriate engineering surface chemistry. All these approaches offer pre-determined particle size range and distribution, hydrophobicity–hydrophilicity balance, porosity, and polymer chain/gel network, drug encapsulation capacity, sustained/stimuli-responsive drug release as a function of formulation characteristics. Table 1 provides a list of various nanoformulations that can preferably encapsulate two drugs and possess sustained delivery to induce synergistic activity. The sections below delineate more about various types of nanosystems that were widely employed for GEM combination delivery.

### 3.1. Liposomes

Liposomes are the vehicles commonly used for drug delivery applications [55]. Liposomal integrity consists of the modulation of lipid structure with a hydrophilic head and hydrophobic tail molecules facing each other in bilayered phospholipids. This is commonly achieved by electrostatic interaction, hydrogen bonding, and Van der Walls forces, and can be modified in aqueous solution [56]. Liposomes (a size range of 50 to 150 nm) are suitable for drug delivery applications and capable of the loading of both hydrophilic and hydrophobic drugs within the core of the formulation and the hydrophobic one between the lipid-based bilayer, respectively. Clinical data on the co-delivery of PTX and GEM show synergistic action. However, co-delivery of PTX and GEM is a challenging task because of the difference in aqueous solubility (PTX is almost insoluble in water) and pharmacokinetic profiles (Abraxane with a half-life of 27 h, mean total clearance of 15 L/h/m^2^ and mean volume of distribution of 632 L/m^2^, GEM with a half-life of 42–94 min, mean total clearance of 30.7–92.2 L/h/m^2^ and mean volume of distribution of 50 L/m^2^) [57,58]. These differences have made it impossible to deliver both the drugs together in an equal ratio, which is important for their synergistic effect. The solution to this problem may be a delivery vehicle which can efficiently encapsulate both drugs within a formulation. Co-encapsulation of dual drugs in the formulation is not always suitable for efficient and sustained drug release [59,60]. Co-encapsulation of GEM + PTX within a single formulation as Lipo-PTX-GEM (LpPG) leads to poor drug release because of the hinderance of PTX on GEM where membrane-bound PTX hinders the diffusion function of the liposome. To overcome this problem, GEM and PTX were formulated separately as LpP (PTX loaded liposome) and LpG (GEM loaded liposome) using DPPC, cholesterol, and DSPE-PEG2000 followed by small volume loading method. Comparative studies were done using LpP and LpG, which provided the synergistic activity of both drugs when given in a scheduled dose and ratio. PTX-GEM incorporation as LpPG did not provide adequate drug release and pharmacokinetic profile but cytotoxicity testing on SKOV-3 human ovarian cancer cells with a separate formulation of LpP and LpG showed better activity. GEM release from LpPG was 37.9% and that from LpG was 66.1%; therefore, the combination treatment of LpP and LpG came up to be more effective than LpPG [60]. High drug dose is the responsible factor for off-targeted cytotoxicity and side effects but Gallo and co-workers, who developed the liposomal-based formulation of DOX in combination with GEM, showed better circulation and extravasation to the tumor sites in cisplatin-resistant human ovarian cancer Xeno-grafts(A2780/CDDP) female athymic mice (HSD: Athymic Nude-nu) thereby confining the drug within the tumor site, which decreases the probability of drug-related toxicity. Combination treatment with a GEM dose of 20 mg/kg and a Liposomal DOX dose of 6 mg/kg was assigned for treatment groups (mice) which were compared with mice under similar GEM monotherapy. All mice receiving this drug combination showed regression in tumor growth with no growth for 2 months. A further decrease in the drug dose to 10 mg/kg GEM and 6 mg/kg liposomal DOX treated in two treatment schedules—one with GEM collaterally treated with DOX-liposome and the other treatment of 24 h GEM treatment proceeded by DOX-liposome treatment—showed almost similar results as obtained by high GEM dose, which suggested that a low drug dose can be substituted to avoid toxicity. The actual mechanism involved in the synergistic activity of GEM + DOX was unclear for the group to depict but targeting different metabolic pathways may be a reason for consideration for the synergistic activity [61]. EndoTAG-1, a cationic lipid (DOTAP and DOPC)-based PTX complex, is a conventional agent used in vascular chemotherapeutic targeting; it was checked for its enhanced anticancer effect in a L3.6pl pancreatic tumor model when combined with GEM. Groups treated with EndoTAG-1 monotherapy showed a significant reduction in tumor growth when compared with control and an EndoTAG-1 dose of 5 mg/kg body weight thrice every week gave the same results as that of a GEM dose of 100 mg/kg body weight twice weekly. When EndoTAG-1 was combined with GEM, it showed a decrease in tumor growth up to 78% as compared to control, which further led towards the investigation of a lower dose of GEM. A decrease of the GEM dose to 50 mg/kg body weight showed decreased antitumor activity to 30% which was again restored by combining EndoTAG-1 with 50 mg/kg body weight GEM, giving a supra additive effect. Tumor volume for the group treated with EndoTAG-I + GEM was significantly less than the control group and the groups with EndoTAG-1 and GEM monotherapy. Inclusive to its antitumor effect, the combinational treatment decreased the occurrence of liver, lymph node and peritoneal metastasis [45]. Poly(HPMA-*co*-MA-GFLG-Gemcitabine-*co*-MA-GFLG-doxorubicin-*co*-MA-TyrNH2) is a construct of a HPMA polymer conjugated with 6.4 wt % GEM and 5.7 wt % of DOX with 1 mol.% tyrosinamide (known as P-GEM-DOX) which exhibited a superior cytotoxicity effect. The efficacy of GEM and DOX was enhanced using this multi-drug targeting formulation without an increase in its level of toxicity. Effect of the formulation on tumor growth was compared using a control group where the results showed that the formulations with a 50% maximum tolerated dose regimen of P-GEM-DOX showed a 50% reduced tumor growth and formulation with 75% MTD showed a reduction in the tumor up to 60%, which was significantly higher than the control group and the groups with a low dose of P-GEM-DOX. There was no significant decrease in body weight of control group and the group with a low dose of P-GEM-DOX with a maximum of 5% loss in the body weight of groups treated with 50% and 75% maximum tolerated dose regimens of P-GEM-DOX [45]. In recent years, a fabricated form of nanoparticles has been much more in use. Thermoresponsive liposomes were introduced to release drugs at the specific tumor site. These liposomes have a phase transition temperature of 42 °C when coated with a suitable polymer which makes the formulation release its therapeutic moiety only when triggered thermally. The normal body temperature of 37 °C does not allow the liposome to avail the drug at normal cellular environments but mild hyperthermia at the tumor site triggers drug release at the specific site; therefore, this modulation provides significant drug availability at tumor sites [45,62]. Layer-by-layer co-loading of gemcitabine and platinum (IV) prodrug nanoparticles were constructed for synnergestic combination [63]. Similarly, a GEM + Cis-based thermosensitive liposome was formulated using DPPC/HSPC/Chol/DSPE-PE2000 polymers in a molar ratio of 100/50/30/6 which favors cellular internalization at 40 °C in BxPC-3 and MiaPaCa-2 cancer cells [63,64], thereby decreasing the off-target effect and systemic toxicity [63]. The reticuloendothelial system (RES) is the main site that liposomes reach after getting administered, from which they get further cleared out from the system. RES is also involved with the bodies innate immune system. Therefore, in spite of liposomes being an important carrier suitable for drug delivery, during the delivery of anti-cancer drugs, liposomes cause the destruction of macrophages as they are part of the body’s innate immune system—this causes an imbalance in the immune system leading towards immunosupression and infection [64,65]. Though liposomes play great role in incorporating dual drugs in the same formulation, helping hydrophobic drugs surpass the lipid bilayer, there are drawbacks associated in their use as any leakage and fusion with the lipid bilayer may cause a loss in the amount of drug required at site of action, which may not provide the desired action. Apart from this, the phospholipids which are used for making liposomes may undergo oxidation and hydrolysis at altered conditions which could cause a loss of the quality of formulation [66].

### 3.2. Nanogels

Nanogels are three-dimensional cross-linked (physical and chemical bonds) network polymeric structures which can offer drug depot and controlled drug release for targeted drug delivery [67]. Nanogels possess a great tendency of water retention and are biocompatible and biodegradable. GemC12-LNC is a nanogel-based nanomedicine synthesized using lauryl and GEM as the chief therapeutic agent. GemC12-LNC is modulated in a way where the lipophilic drug (PTX as a model) can be incorporated in the oily core whereas the hydrophilic one gets incorporated inside the aqueous phase. PTXGemC12-LNC treatment to GL261 and 9L cell lines showed its enhanced therapeutic activity tested through the invasion, proliferation, and the aggressive aspects of cancer [68]. Amphiphilic block polymers of PNIPAM-*b*-PNAM-*b*-PNBOC were used for the formulation of thermo-responsive hydrogel with a PNBOC polymer to be around 68 nm with a spherical structure to contain the photo responsive moiety. Being a photo and thermosensitive formulation, it can control the hydrophilic and hydrophobic drug release. Besides these considerations, the effect of UV light and polymer ratios were also concerns for the effective drug release of GEM and DOX. Drug release was much higher in the presence of UV radiation, than in the absence of UV radiation. In higher ratios and concentrations of polymers, drug release was decreased despite increased UV radiation therefore, it is important to maintain a constant ratio of PNIPAM-b-PNAM-b-PNBOC in the presence of UV radiation [54]. GEM and cisplatin are standard drugs and widely used for the clinical treatment of cancer. The problem associated with them is their toxicity and non-targeted effect. Loading Cisplatin within nano gel formulation (PEG170-b-PMA180 and Mal-PEG-NH2 conjugated with TKH2 antigen, a specific antigen aberrantly expressed in PanCa) helped in improving the PK of cisplatin. A higher amount of platinum reached the cells expressing Sialyl Tn antigen-specific with TKH2-cisplatin nanogel binding. The dose of cisplatin in GEM 79 combined TKH2-cisplatin hydrogel came up to be 40-fold less than the dose of cisplatin when used as monotherapy. GEM and TKH2-cisplatin hydrogel when given as monotherapy was responsive to primary tumor suppression which was further suppressed by GEM with TKH2-cisplatin hydrogel. The tumor-suppressing effect was better than the non-targeted formulation of GEM + IgG-cisplatin hydrogel but was not statistically significant enough. A metastatic check on other organs revealed that there was no significant metastasis to other organs in the groups treated with GEM + TKH2-cisplatin hydrogels. Therefore, GEM + TKH2-cisplatin hydrogel as a formulation is an efficient delivery vehicle to sensitize platinum within cancer cells which is guided by GEM-added combinational therapy and TKH2 antibody conjugation [40]. Use of nanogels have not only enhanced the delivery of GEM in combination with other drugs but has also helped in generating other routes of GEM administration. GEM is a drug which is preferably administered through the IV route as there are limitations to its oral route of administration such as poor GI permeability and rapid deamination of GEM. Preparation of the polymeric nanogel was done using PVA polymer grafted with cholesterol and incorporated with GEM by the process of co-evaporation. Mia PaCa-2 and Capan-1 cells were used for in vitro studies and the tumor inhibitory studies were carried out in tumor xenograft female nu/nu mice model. Nanogel encapsulated GEM and Floxuridine showed 3 to 25 times higher cytotoxicity against PanCa cells than the free drug. A GI permeability check was also carried using the Caco-2 cell model which showed permeability constant of 7.5 times more for GEM encapsulated PVA nanogels than GEM alone. Conducted studies showed effective drug release triggered by enzymatic activity within cancer cells and results from animal experiments. Though the delivery of chemotherapeutic drugs using nanogels is advancing swiftly because of its ability to be fabricated according to changes in pH and temperature, the synthesis and removal of solvent during the formulation is quite expensive and, apart from this, the presence of any monomer or surfactant can add additional toxicity to the formulation.

### 3.3. Micelles

Polymeric micelles possess a unique shell-like structure. For drug delivery purposes, micelles are modified copolymers with a hydrophobic core and a hydrophilic shell. Mondal et al. [40] showed the impact of an increased GEM dose which leads to GEM-related drug resistance impacting the therapeutic activity of GEM. GEM and miR-205 have been formulated using PEG-*b*-PCC-*g*-GEM-*g*-DC-*g*-TEPA and conjugated with the EGFR targeting Cetuximab antibody. This formulation is efficient to target GEM at pancreatic tumor sites. Both in vitro studies on MIAPaCa-2 and in vivo studies on MIAPaCa-2 orthotopic mouse models showed enhanced drug uptake in the group treated with formulations containing Cetuximab as the targeting antibody than the control group. Mice showed low tumor volume when treated with the GEM-miR-205-Cetuximab conjugated formulation with no alteration in body weight, suggesting negligible drug-based toxicity [40]. GEM and PTX, being standard therapy against carcinoma, have faced problems during co-delivery (which has been discussed in previous sections). An FA-PEG-VE (folic acid and tocopherol conjugated PLGA-based micelles) formulation demonstrated significant inhibitory effects against A549 cells when GEM-PTX were incorporated. A more synergistic drug effect was observed in vivo and pharmacokinetic analysis when the formulation was modified with tocopherol [45]. Though chemotherapy aims to eradicate tumor and cancer cells, chemotherapy does not function efficiently to eradicate the slow-growing cancer cells also known as cancer stem cells [69]. The use of mixed micelle-based nanoparticles (PEG-*b*-PAC and PEG-*b*-PUC through hydrogen bonding and ionic interaction) shows a high drug loading capacity with an ability to incorporate GEM and Phenformin, exhibiting good activity against cancer stem cells [70,71]. The activity of Lauroyl-GEM (Gem-C12) and honokiol have already been reported as a treatment where GEM helps in terminating the elongation of DNA chain leading to apoptosis, and honokiol as an inducer of apoptosis. Thus, nanoparticle formulation of the Gem-C12 and HNK combinational therapy was able to reach the targeted site of action [72,73]. CD44 can be a useful target in cancer treatment because of its aberrant overexpression in cancer cells. Hyaluronic acid (HA) is yet another source which shows an affinity with CD44 therefore, HA in conjugation with CD44 can be used as a formulation to avail HA in the tumor-specific site. Formulation of bile salts/phosphatidylcholine mixed micelles (BS/PC-M)-HA encapsulated with Gem-C12 and HNK utilized the EPR effect to provide drugs to the targeted cancer cells [74]. Micelles have come up as a great source for co-delivering GEM with other chemotherapeutic drugs, but micelles have a problem of poor drug loading efficiency, poor structural stability when used in-vivo, and the micelles themselves are sometimes unable to interact with the malignant tumor cell and therefore cannot avail the drugs at the targeted site of action [75].

### 3.4. Albumin Nanoparticles

Albumin bound paclitaxel (Abraxane^®^ or nab-PTX) is a formulation which has overcome the limitations associated with the paclitaxel drug with a satisfactory solubility in water. Paclitaxel, when combined with Gemcitabine, has shown an increase in the level of Gemcitabine within tumor cells by decreasing the activity of cytidine deaminase, an enzyme with an ability to decrease Gemcitabine metabolism. nab-PTX possesses an ability to distort the stroma of PanCa cells which assist in activating angiogenesis, thereby increasing circulation, making it easier for GEM to reach targeting sites. Despite these advantages, this drug combination has shown significant toxicity which demands efficient studies and trials for drug approval [49]. Phase I and II clinical trials were conducted on GEM and nab-PTX to determine the maximum tolerated dose of GEM and nab-PTX in treating PanCa, inclusive of its response towards positron emission scan analysis which helps in determining the alteration in the pancreatic stroma, influencing drug uptake. Successful clinical trials have fixed a standard combinational dose of 1000 mg/m^2^ GEM with 125 mg/m^2^ of nab-PTX given for a 3-week period in repetition every 4 weeks. Overall survival, total response rate, and the maximum tolerated dose obtained from these studies were quite higher. According to their preclinical study, the nab-PTX is intended for the destruction of the peritumoral desmoplastic stroma, therefore, they increased the intra-tumoral concentration of GEM within the treated mice group used for their study. Although these studies gave a direction in using this combination effectively against PanCa, a randomized phase III clinical trial was much needed to bring up this combinational therapy as an acceptable therapy for the significant treatment of PDAC [76]. Phase III trials were conducted by employing a total of 861 patients which provided positive data showing greater efficacy of the nab-PTX + GEM combination over GEM monotherapy. Overall survival was 8.5 months in the nab-PTX + GEM group whereas was 6.7 months in the Gemcitabine group, with a survival rate of 35% in the nab-PTX + GEM group and 22% in the GEM group at one year of drug administration, with 9% in the nab-PTX + GEM group versus 4% in GEM group after two years of drug administration. The median progression-free survival was 5.5 months in the nab-PTX + GEM group, as compared with 3.7 months in the Gemcitabine group with the response rate of 23% in nab-PTX + GEM group versus 7% in the GEM group [77]. OMP-54F28, used in combination with nab-PTX and GEM on patients with previously untreated stage IV PanCa had a positive phase I clinical trial (NCT02050178).

### 3.5. Multifunctional Nanoparticles

Apart from chemotherapy, other types of physical methods (such as hyperthermia, photothermal therapy) can enhance chemotherapy activity. Hyperthermia plays a vital role in enhancing the chemotherapeutic efficiency of drugs by alleviating the temperature of cancer cells, and, as a result, enhancing their sensitivity towards chemotherapeutic drugs. GEM + Cis-based thermosensitive liposomes have been formulated using DPPC/HSPC/Chol/DSPE-PEG2000 polymers. These liposomes when modified with polymer to lead to multi-functional nano formulation have a phase transition temperature of 42 °C which makes the formulation release its therapeutic moiety only when triggered with hyperthermia [64]. The normal body temperature of 37 °C does not allow the liposomes to avail the drug in a normal cellular environment but mild hypothermia at the tumor site triggers drug release at the specific site; therefore, this modulation provides significant drug availability at tumor sites [78]. Non-damaging mild hyperthermia assists as an external stimulus to surpass the cellular barrier and avail the drug at tumor-targeted sites [79]. Mild hyperthermia and GEM Lip combined therapy are used where MHT from gold nanorods have been designed in a way to enhance the delivery of particles with a size less than 100 nm. A 3-fold increased delivery of GEM Lip at the tumor site with a diameter of 80 nm, MHT with GEM Lip showed a 4-fold reduction in tumor growth which was due to the enhanced accumulation of GEM within the tumor cells [80]. To enhance the half-life of GEM, maintain its active metabolite, decrease the dose intake and drug-related toxicity, special carriers are needed which can enhance overall GEM efficacy [64,81]. GEM-based magneto liposomes have been formulated as a superior carrier for GEM because it involves the least use of organic solvents during drug incorporation and is therefore suitable for in vivo applications. Magnetite-based nanoparticles (DPPC/cholesterol) were also used for encapsulating GEM by the process of coprecipitation. Under the influence of a magnetic field, the formulation showed 70% drug release after 5 min of exposure. The formulation had the desired size and zeta potential to be used as a treatment against cancer. Therefore, magnetic properties of the formulation provided room to enhance temperature at drug targeting sites which enhances GEM delivery. Techniques which can help to detect the accumulation of nanoparticles can be important to track down the organ being targeted by the particles, which is helpful in drug release from nanoparticles. These abilities help in modifying drugs according to the obtained toxicity and off-target effects [82]. Gadolinium with low molecular weight enhances the T1 weighed MRI contrast of tissues which makes it easy to image tissues targeted with Gadolinium as an imaging agent [83]. A formulation containing a combination treatment agent as well as the imaging agent designed by Li et al. [84] utilizes magnetic resonance to image tumor sites and confine the GEM drug at image-guided sites, enhancing the retention time of GEM. The formulation came up as an efficient agent to show significant drug release at the tumor-specific site and MRI helps in determining the high resolution of tumor sites. Gadolinium, an imaging agent, and GEM, a therapeutic agent, have been prepared as a self-assembled formulation where the formulation was PEGylated to enhance its circulation time within the tumor responsive sites. The efficacy of Gadolinium and GEM loaded nanoparticles showed the highest (72%) tumor inhibition and delay in tumor growth than other comparative groups. Toxicity studies suggested the neutral nature of the nanoparticles. Therefore, Gadolinium and GEM-based nanoparticles were efficient enough to inhibit tumor growth, but they possessed the significant problem of early clearance, which still needs to be addressed [84]. Gold-based nanoparticles are easy to synthesize with variable shapes and sizes in addition to high biocompatibility and low toxicity, the surface property of gold nanoparticles can be easily modified and modulated using targeting moieties [85]. Compared to other imaging agents, gold nanoparticles tend to be a more effective agent for X-ray which makes them a better agent for CT imaging [86]. Still, there are limitations added with the benefits of using multipurpose nanoparticles. The tumor microenvironment provides a major hinderance to the nanoparticles carrying drug cocktails. The physiological barriers present within the tumor microenvironment such as the dense collagen network, hyper vasculature and high fluid pressure are the prime sources which hinder the proper circulation and availability of nanotherapeutics at the targeted site of action [87].

### 3.6. Targeted Nanoparticles

**Figure 4:**
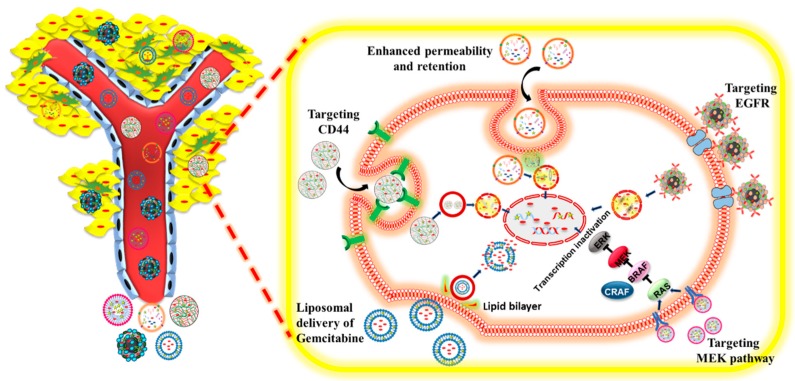
Passive (EPR) and active (ligand/receptor-based) targeting of Gemcitabine-based co-formulation.

Nanoparticles have been functionally modified to come up with chemotherapeutic formulations with significant pharmacokinetic property, non-toxicity, and biocompatibility. Liposomes, solid nanoparticles, polymer conjugates, micelles and etc. have come up as an emerging source for drug delivery; but to get established clinically, various trials are still needed for validation. Therefore, in a broader way liposome and polymeric nanoparticles are the formulations which are being clinically used for drug delivery in a broad sprectrum of different drug and drug combinations [88]. However, most of the tabulated and FDA-approved nanoparticle-mediated deliveries primarily rely on the EPR effect and accumulate in solid tumors due to leaky tumor vasculature. Historically, such delivery approaches offer the advantage of minimal to medium levels of injected doses of drugs, up to 5 to 10%. Active targeting mechanisms always exploit and seek safe homing through a target receptor or membrane protein that typically overexpress on tumor cells. To achieve an increase in tumor delivery by site-specific and active delivery by attaching active moieties that recognize cancer cells precisely, such decorative or conjugated nanoformulations have the tumor-homing potential which can enhance drug distribution in tumors (Figure 4). Mucin is expressed by various epithelial cells, but when when we talk about PanCa, mucin is abberantly expressed by pancreatic cancer cells which positively stimulates the growth, proliferation, invasion, and differentiation [89]. This has made mucin a prospective tumor marker which facilitates the targeting of anticancer therapeutics. MUC13 as tumor marker in PanCa has been studied [90]. This study demonstrated high MUC13 expression in HPAFII and Capan-I in PanCa cells compared to normal cells. Data was also presented to show that MUC13 plays significant role in tumor progression by facilitating invasion, metastasis, and cellular motality with a positive influence of tumorogenic pathways [90]. MUC4 is a class of mucin which, when expressed during a PanCa condition, caused resistance towards GEM and other neucleoside-based drugs [91]. Another study involving the targeting of the mucin-based biomarker has been done as antibody guided GEM targeting by Urey et al. [37] utilizing the Anti-MUC4 antibody and IgG to formulate iGem Lip which involved covalent linking of the IgG antibody and further coupling which thiolated anti MUC4 at 1:1000 molar ratio of the IgG present in the formulation. Obtained results showed significant binding of the iGemLip formulation with MUC4 positive capan-1 cells in a dose dependent manner when compare with GemLip alone. Cytotoxic studies were also significant enough to depict enhanced cytotoxity of iGemLip when compared with GemLip and GEM alone. Therefore, this study brings an impotant targeting-based delivery of GEM using mucin markers but in vivo studies are still required to provide more concrete evidence for the formulation. Some of the nanoparticle-based delivery approaches which can be used in a combination with GEM specific towards receptor targeting are mentioned in Table 2.

## 4. Conclusions and Future Perspectives

Co-delivery mechanisms with GEM can be an effective tool for enhancing the survival rate of PanCa patients and decreasing the problems associated with dual drug co-administration. The recent research conducted with the co-delivery of GEM has shown promising outcomes such as the ability to decrease individual drug doses, incorporating drugs of different physicochemical properties together, which warrants further investigation. Although there are certain limitations with co-delivery techniques such as overcoming the barrier of the physical/chemical features of two different types of specific drugs or therapies when applied together, it is imperative to find alternative solutions to improve the efficacy of the combination. Also, uniform drug loading capacity, the stability of the drug combination, and higher uptake capabilities should also be taken into account. The availability of various drug delivery options has opened up scope to work on the approach of how the delivery vehicle can be fabricated in a better way to limit the curbs associated with the delivery of GEM with other drug cocktails. Combining both hydrophilic and hydrophobic drugs into a single system can prove to be an efficient technique for the treatment of PanCa patients. Based on the current research and success profiles of various co-delivery techniques, significant GEM-based co-delievry formulations based on polymeric micelle has been noticed; therefore, further work on these nanocarriers could help to promote polymeric micelles as an excellent source of GEM-based co-delivery. This can also help in designing polmeric carriers in such a way that it can have the properties of different carriers altogether such as liposome and hydrogel properties within itself, which can make polymeric micelles more efficient in delivering GEM and other drugs; therefore, these carriers could be nearer to clinical trials than the other carriers which are being studied now although more advanced studies are required in this direction to reduce the limitations which are still present with the different nanocarriers, which would possibly enable more concrete findings to proceed to clinical trials.

## Figures and Tables

**Table 1:** Various types of nanosystems at a pre-clinical step for optimizing and inducing the synegistic action of drugs in pancreatic cancer.

Nano-Systems	Drug Conjugate	Characterization	Utilization
(Micelles) GEM-PL	GEM-methoxy poly(ethylene glycol)-poly(lactic acid) (GEM-PL)	Particle size 112.2 ± 1.86 nm, zeta potential, 5.2 ± 1.26, encapsulation efficiency 92.5 ± 3.26 loading efficiency of 14.6 ± 1.29 and PDI(Poly dispersity index) 0.118.	In-vitro study was done using human colon cancer cell line HT29 and in-vivo studies for anticancer efficacy was done by injecting HT29 (5 × 10^6^ cells) subcutaneously into right flank per mice and when tumor volume reached 100 mm^3^, 5 mg/kg fixed drug dose was administered 3 times for 12 days. In-vivo studies depicted improved blood circulation time with greater accumulation of the drug in tumor site with significant tumor regression. Formulation killed HT29 cells at a time dependent manner [39]. This study provides a valid reason required for further clinical research.
(Micelles) C225—micelle-GEM-miR-205	GEM-miR205-EGFR targeting Cetuximab antibody (C225)	Unmodified micelles containing GEM and miR-205 had the size of 76.6 ± 6 nm and zeta poteintial of 4.7 ± 1.65 mV. C225 conjugation was checked with standard BSA which was found to be 510 ug/mL C255 micelle 30% *w*/*w* for 10 gm polymer.	In-vitro studies were conducted using MIA PaCa-2 and in-vivo studies such as biodistribution and efficacy studies were done on NSC mice using an orthotopic tumor model where mice were implanted with GFP-transfected MIA PaCa-2 cells. In-vivo studies were conducted using a orthotopic pancreatic tumor model in 6 week old NSG mice injected with GFP transfected MIA PACA-2 cells [40].
(Micelles) PHC-GEMC18	poly(ethylene glycol) (PEG) conjugated hydrophobic stearic acid derivative (C18) bonded through acid sensitive hydrazine bond-GEM	PHC size of 21.6 ± 0.6 nm zeta of 3.3 ± 1.1 mV PHC3%(GEM) size of 9.6 ± 3.7 nm zeta of 2.7 ± 1.2 mV PHC5%(GEM) size of 5.0 ± 0.1 nm zeta of −2.0 ± 0.7 mV. PHC10%(GEM) size of 9.7 ± 1.3 nm zeta of −2.2 ± 0.9 mV.	B16-F10 tumor was grown on C57BL/6 mice by subcutaneous inoculation in the right flank followed by treatment after day 6 of inoculation. As compared to GEM alone which is because of the increase in GEM18 accumulation [41].
(Micelle) GE11-PEG-PCD/mPEG-*b*-PCC-*g*-GEM-*g*-DC	G11 peptide of GEM conjugated with poly (ethylene glycol)-block-poly (2-methyl-2-carboxyl-propylenr carbonate-graft-Gemcitabine-graft-dodecanol micelle	Particle size of 26 ± 3 nm with a polydispersion index of 0.27 [42].	MIA PaCa-2 cells have been used for the in-vivo studies and 6–8-week-old athymic nude mice were used for a orthotopic pancreatic cancer model for studying efficacy of the micelles after intraperitoneal injection of D-luciferin for bioluminescence studies. Increase in GEM delivery was seen up to 2.5 folds with an enhanced circulation half-life and EPR effect facilitating extravasation of micelle loaded drugs within the tumor microenvironment [43].
(Micelle) DTX-PEG-GEM	Docetaxel-Polyethylene-glycol-GEM	Particle size of 124.2 ± 5.7 nm and PDI of 0.132 ± 0.03 with critial micelle concentration range of 5–10 × 10^−3^ mg/mL.	In-vitro studies were done using MCF-7 and MDA-MB-231 for checking cellular internalization and uptake, In-vivo study used female Sprague dolly rats for carrying forward the pharmacokinetic and toxicity studies. Clathrin mediated endocytosis with 4.8 fold higher AUC value as compared to Gemzar alone was observed with a noteworthy decrease in tumor volume, increase in total survival, and reduction in hepatic, nephron, and hemolytic toxicity when administered with DTX-PEG-GEM nanoparticles [44].
(Micelle) P-GEM-DOX	Poly(HPMA-co-MA-GFLG-GEM co-MA-GFLG-DOX-co-MA-TyrNH2)	Molecular weight = 23.5 kDa, PD = 1.6, GEM = 6.4 wt %, Dox = 5.7 wt %, tyrNH2 = 1.0 mol %.	Dunning AT1rat prostate carcinoma cells were used for the in-vitro studies which involved drug release, cytotoxicity, and efficacy study of the formulation. In-vivo studies were conducted on male Copenhagen rats present with subcutaneous tumor. Enhanced circulation time with selectivity and localization at tumor-specific sites was seen with induction of apoptosis and inhibition of angiogenesis. In-vivo efficacy of P-GEM-DOX < free GEM, though co-conjugation enhanced in vitro efficacy [45].
(Micelle) GEM-LEMPs-DNA	PEGylated lipid bilayer cationic ε poly lysine co-polymer with GEM-(si-HIF1α)	Particle size of 60 nm with a hydrodynamic diameter according to DLS study, size was 141.8 nm zeta potential of GEM-LEMP-DNA was −34 mV, encapsulation efficiency of 42%.	Serum stability, cytotoxicity, PCR, and immunohistology studies were done using Panc-1 and B-16 melanoma cells. The in-vivo studies were conducted on female BALB/c mice to check the antitumor activity of the formulation. Formulation caused effecting silencing of HIF1α via siRNA and reduced drug-related resistance. The lipid layer protects si-HIF1 α from degradation thereby maintaining the integrity of the particle and preventing leakage of GEM [46].
(Micelle) GEM-C18-PLGA-MP	Stearoyl GEM incorporated within PLGA with surface functionalization with human serum albumin	GEMC18 content was found to be 488.9 ± 35.7 μg/mL with PLGA content of 1.71 ± 0.21 mg/mL corresponding to 285 ± 56 μg/mg or 28.6% ± 5.6% (*w*/*w*) of particle matrix, prodrug release of 13.2% ± 1.8% was seen after 5 days incubation.	SV-HUCC-1 normal urinary bladder cell was considered as control and urothelial cancer cell line 5637 and HT-1376 were used for a cell viability assay, studies for determining metabolic activity and for checking the cyto adhesive properties of formulation. GEMC18, when conjugated with PLGA microparticle, avoided intracellular drug activation thereby maintaining drug stability and covalent modification of the polymer with human serum albumin, led towards the enhanced binding capacity of the formulation with urothelial cells [47]. In-vivo studies using an animal model is still a requirement to confirm the pharmacokinetics of the formulation.
(Micelle) FA-PEG-GEM-NPs	Folic acid conjugated GEM loaded surface modified chitosan nanoparticle	Particle size determined to be 184.3 ± 12.47 nm with a PDI of 0.22 ± 0.07 zeta potential of 21.1 ± 1.18 mV and encapsulation efficiency of 37.2% ± 2.2%.	In-vivo cytotoxic studies were done using lung epithelial cancer cell line A549 for a cytotoxic assay, drug release and cellular uptake. Balb/c mice were used to conduct the pharmacokinetic study. Significant cytotoxicity showed while GEM being delivered through nano formulation when treated to A549 cells showed more effective cellular internalization than free GEM [48].
(Metal-based) IONPs	GEM-siRNA-iron-oxide nanoparticles	Particle size of 80 nm.	Iron oxide was profoundly conjugated with CD44v6 targeted PanCa and GEM-siRNA conjugation with siBmi-1 oncogene to give multifunctional nanoparticle scFv-GEM-siBmi-1-NPs an in vivo anti-tumor synergistic activity [49].
(Metal-based) MIL-100 Nano-MOFs	Metal-organic framework of iron III trimesate nanoparticles-phosphate GEM	Encapsulation efficiency of phosphated GEM = 30.7% ± 0.8% which was almost 98%.	GEM-MP loaded NanoMOFs were studied on PANC-1 cells in a phosphate devoid medium with 50% of encapsulated drug released within 1 min after administration which stayed for 20 h [50].
(Metal-based) PS1-EPSMOs-GEM	Tetrasilylated porphyrin-ethylene periodic mesoporous organosilica nanoparticles	PS1-EPSMOs mean diameter (TEM) = 447 nm, zeta at pH 5.5 = −30 mV; zeta at pH 7.4 = −34 mV.	In-vivo delivery of drug was done on MCF-7 breast cancer cells. The porous structure provided high loading capacity and the addition of the porphyrin group provided photosensitivity to the nanoparticle [51]. In-vivo studies are yet to be done for confirming the significance of this formulation.
(Metal-based) AuNC@BSA-MSN-GEM-DOX	Gold nanocluster bovine serum albumin clustered with mesoporous silica added with 32% GEM and DOX combined with albumin, attached electrostatically to formulation	Nanoparticle size = 150 nm, gold-protein conjugate zeta potential of −38 ± 1 mV for MSN-AuNC@BSA + DOX + GEM with AuNC content of 2.10 ± 0.23 and BSA 15.90 ± 1.80.	In-vitro study for the formulation was done using A549 lung cancer cells and biodistribution of formulation was seen in nude mice being previously injected subcutaneously with MIA-PaCa-2. Dual loading of GEM + DOX was 72 wt % which was four times higher than previous reports with less than 4% leakage of the loaded drug after a week in blood serum [52].
(Metal-based) PTX-GEM-LB-MSNNP	Lipid layer mesoporous-silica nanoparticle loaded with PTX and GEM	Hydrodynamic partilc size of 101 nm in saline and 112 nm with zeta potential of of −27.2 mV and −5.4 mV in saline plus 5% serum condition.	Cytotoxicity study, expression of cytidine deaminase and heme oxygenase via Western blot was done using PanC-1 cells and for the in-vitro studies, these cells were transfected with luciferase and implanted to grow subcutaneously within xenograft nude mice. Co-delivery of a dual drug caused enhanced phosphorylation with an increase in DNA-GEM interaction up to 13 fold and decreasing inactivated deaminated metabolite up to 4 folds producing synergistic codelivery of GEM and PTX [53].
(Metal base) GEM-Au DENPs/miR-21-inh	Ultrasound targeted microbubble-based dendrimer entrapped gold particle-Gemcitabne-miR-21 inhibitor	Mean particle size obtained was 154–276 nm with a surface charge range of 11–33 mV.	SW1990 cells were used to check the effect of the formulation on cytotoxicity, female athymic Balb/c mice were used to check the antitumor activity of the formulation. Uptake and apoptosis. The apoptosis percent of GEM–Au DENPs/miR-21i group (20.87% ± 0.81%) and GEM–Au DENPs/miR-21i + U group (25.43% ± 0.60%) which came up to be much more than the free GEM group (10.50% ± 0.56%) [40].
(Hydrogel) PNIPAM-*b*-PNAM-*b*-PNBOC	poly(*N*-isopropylacrylamide)-*b*-poly (4-acryloyl morpholine)-*b*-poly(2-(2-nitrobenzyl)oxy) carbonyl) amino)ethyl methacrylate)-GEM-Doxorubicin	Average hydrodynamic diameter was determined to be 68 nm. TEM observation gave spherical nanoparticles.	Extra micellar aqueous phase and the hydrophobic micellar core of formulation helped in the incorporation of a hydrophilic and hydrophobic drug [54]. This work showed the synthesis and characterization of formulation which needs follow up with in-vivo and in-vitro studies.

**Table 2:** Receptor-based targeting of Gemcitabine-based co-formulation designed using various polymers.

Formulation	Target/Ligand	Process of Conjugation	Outcome
MPDNCs Poly(l-lysine) carboxylate PTX(PLL-PTX) + Hyaluronic acid conjugated GEM	CD44	Electrostatic attraction between PLL-PTX and conjugation between HA-Gem through hydrolysable linkers.	Biliary cancer cells HuCCT1 and SCK have been used to check the targeting efficiency and therapeutic efficacy of the formulation. Xenograft Balb/c nude mice were used for checking in vivo drug efficacy. Cellular uptake of MPDNCs induced synergistic apoptosis [92].
GEM-AuNPs-C225	EGFR	Incubation of AuNPs with 2 μg/mL C225 for 1 h at pH 7.8 proceeded by 1 h incubation with GEM 5 μg/mL.	Screening of PanCa cells such as PANC-1, AsPC-1, and MIA PACA-2 with EGFR expression enhanced the targeting efficiency of formulation with a significant reduction in cell proliferation and tumor growth in orthotopic nude mice injected with GFP transfected AsPC-1 cells [93].
EM-gold nanoparticle	Plectin-1	Pyrimidine group within GEM provides free NH_2_ group where Gem has an ability to bind with the gold nanoparticle via electrostatic force of interaction.	Surface modulated GNPs with peptides used for plectin 1 targeting and conjugation with GEM showed higher cytotoxicity in AsPC-1 and PANC-1 cell lines with a significant in vivo antitumor efficacy of formulation when given via tail vein in female Balb/c mice with xenografted pancreatic tumor [94].
ATF-IONP-GEM (Amino terminal fragment-iron oxide nanoparticle-GEM)	Urokinase plasminogen activator receptor	Iron oxide nanoparticle are conjugated with amino terminal fragment peptide of uPA receptor domain through lysosomal cleavable tetra peptide linker.	In vitro studies to check drug cytotoxicity, drug targeting in PanCa cells was done using MIA PaCa-2. The anti-tumor activity was checked in-vivo with the help of MIA-PaCa-2 implanted xenograft model in nude mice. Drug dose given twice weekly. Endocytosis through receptor-mediated approach enabled the release of GEM within the cells which helped in intensifying MRI of the tumor and the presence of lysosomal cleavable bonds prevented the formulation from enzyme-based degradation [95].
GEM-Chitosan-Carbopol-MNPs	Folate receptor	Surface conjugation of Poly acrylic acid polymer with chitosan forming multilayer shell with a surface conjugation with folic acid.	PLC-PRF-5, DLD-1, and MDA-231 cell line respective to different cancers were used to carry on the in-vitro studies to depict cytotoxic activity of the formulation. Additional studies were also conducted to check for folate expression within cell. Targeting of folate receptor increased the chance of particles to surpass the cell membrane and make GEM available at tumor sites [96]. This study requires animal work for further validation of the formulation.
Gem targeted TGFβi-MSNP	β-Kinase receptor	Co-precipitation method involved. PEI coating above the MSNP provide great number of non-complex hydrogens which gets attached to the nitrogen atom present in TGF B inhibitor LY364947 via hydrogen bonding.	Endothelial cells, human microvascular endothelial cells and human smooth muscle cells were used to mimic stromal environment and BxPC3 cancer cells were used for in-vitro studies and for implanting mice xenograft. Tumor bearing mice were injected IV. Formulation decreased vascular pericyte coverage by inhibiting TGF β pathway which is caused by LY364947 group of the formulation thereby enhancing efficient uptake of GEM-based IV liposomal formulation coming up as a two-way drug delivery approach [97].
GEM-C18-PEG-DSPE/TPGS	EGF-receptor	Stearic acid conjugated with GEM further been incorporated within PEG-DSPE/TPGS micelles where GEMC18 loaded micelles where prepared using solvent evaporation.	Human BxPC-3 were used to check the proliferation and cellular uptake of the formulation. Antitumor and pharmacokinetic studies were performed on mice injected with BxPC-3 intraperitoneally in the right flank. Formulation avoided GEM deamination which was noticed in free GEM leading towards enhanced GEM circulation time and 3-fold increased GEM concentration in tumor cells [96].
(GMP + VEGF)-LCP-AA	Sigma receptor	Phosphate group present in GEM interacts with calcium during the preparation of micro emulsion leading towards encapsulation of GEM and VEGF siRNA with a surface modification with PEG which increases particle retaining time within body.	30–40% greater tumor inhibition with 8-fold reduced proliferation and decreased tumor microvessel density as compared to alone VEGF and GEM treatment was observed in H460 tumor induced mice with treatment given as IV injection, in vitro cytotoxic studies conducted using H460 non-small lung cancer cell. Multiple nucleic acid incorporation and targeting of sigma receptors found extensively on overexpressing cells [98].
Folic acid conjugated GEM loaded chitosan nanoparticle	Folate receptor	Normal conjugation and centrifugation process were utilized in synthesis of FA-conjugated and PEGylated GEM-NPs.	Significant cytotoxicity showed while GEM being delivered through nanoformulation when treated to A549 cells showed effective cellular internalization than free GEM. Balb/c mice were used to conduct the pharmacokinetic study with formulation injected through lateral tail vein [48].

## Abbreviations

PanCa	Pancreatic cancer
PDAC	Pancreatic Ductal Adenocarcinoma
GEM	Gemcitabine
5-FU	5-Fluorouracil
PTX	Paclitaxel
CIS	Cisplatin
OXA	Oxaliplatin
IRN	Irinotecan
ERL	Erlotinib
CAP	Capecitabine
DOX	Doxorubicin
LEU	Leucovorin
MIT	Mitomycin
CET	Cetuximab
BEV	Bevacizumab
ABL	Ablifercept
SUN	Sunitinib
TRA	Tramitinib
PIM	Pimasertib
dFdCDP	Difluorodeoxycytidine Diphosphate
GEM-MOFs	Gemcitabine Loaded Metal Organic Frameworks
